# Timing of repair and mesh use in traumatic abdominal wall defects: a systematic review and meta-analysis of current literature

**DOI:** 10.1186/s13017-019-0271-0

**Published:** 2019-12-17

**Authors:** Steffi Karhof, Rianne Boot, Rogier K. J. Simmermacher, Karlijn J. P. van Wessem, Luke P. H. Leenen, Falco Hietbrink

**Affiliations:** Trauma Surgery Department, University Medical Centre Utrecht, PO Box 85500, 3508 GA Utrecht, The Netherlands

**Keywords:** Traumatic abdominal wall defect, Traumatic defect, TAWD, Traumatic hernia

## Abstract

**Background:**

Traumatic abdominal wall hernias or defects (TAWDs) after blunt trauma are rare and comprehensive literature on this topic is scarce. Altogether, there is no consensus about optimal methods and timing of repair, resulting in a surgeon’s dilemma. The aim of this study was to analyze current literature, comparing (1) acute versus delayed repair and (2) mesh versus no mesh repair.

**Methods:**

A broad and systematic search was conducted in PubMed, EMBASE, and the Cochrane Library. The selected articles were assessed on methodological quality using a modified version of the CONSORT 2010 Checklist and the Newcastle-Ottawa scale. Primary endpoint was hernia recurrence, diagnosed by clinical examination or CT. Random effects meta-analyses on hernia recurrence rates after acute versus delayed repair, and mesh versus no mesh repair, were conducted separately.

**Results:**

In total, 19 studies were evaluated, of which 6 were used in our analysis. These studies reported a total of 229 patients who developed a TAWD, of whom a little more than half underwent surgical repair. Twenty-three of 172 patients (13%) who had their TAWD surgically repaired developed a recurrence. In these studies, nearly 70% of the patients who developed a recurrence had their TAWD repaired primarily without a mesh augmentation and mostly during the initial hospitalization. Pooled analysis did not show any statistically significant favor for either use of mesh augmentation or the timing of surgical repair.

**Conclusion:**

Although 70% of the recurrences occurred in patients without mesh augmentation, pooled analysis did not show significant differences in either mesh versus no mesh repair, nor acute versus delayed repair for the management of traumatic abdominal wall defects. Therefore, a patient’s condition (e.g., concomitant injuries) should determine the timing of repair, preferably with the use of a mesh augmentation.

## Background

Blunt traumatic abdominal wall defects (TAWDs; also known as traumatic abdominal wall hernias, TAWHs) are uncommon; its reported prevalence is less than 1% after blunt abdominal trauma [1–4]. The mechanism of injury involves a sudden and large impact—such as a seatbelt which digs into the abdomen due to a sudden deceleration after a car collision—leading to shear stress and an elevated intra-abdominal pressure, eventually disrupting the abdominal wall [5–8].

The rarity of TAWD detection in trauma care is mostly due to the fact that less than 50% of all traumatic abdominal defects present with classical symptoms such as reducibility [9–11]. Moreover, they are often masked by superficial injuries such as hematomas or small skin defects [12, 13]. Ultimately, serious concomitant injuries are prioritized at primary care, and there is often no time for scrutinizing the abdominal wall [4, 8, 14]. Most patients who are brought to a level I trauma center have concomitant (intra-abdominal) injuries requiring surgery.

Although TAWDs are rare, the detection rate has increased over the past few years due to a more widely availability and more liberal use of computed tomography (CT) scans in trauma care [1, 3]. Owing to this increase in reported incidence, the debate about the clinical significance and management of TAWDs is highly relevant. However, there is a lack of comprehensive literature on (the management of) TAWDs; it is mainly limited to case reports and a few case series and reviews [3, 13, 15]. Moreover, these publications describe a variety of management strategies, including differences in methods of repair such as mesh use [1, 3, 4, 13, 16–20]. Ultimately, the repair of TAWDs is not standardized. Therefore, when a TAWD is diagnosed, many surgeons are still confronted with a dilemma and the therapeutic strategy is the result of personal experience and insight [1, 5, 17, 21].

The aim of this study was to analyze current literature on the management strategies and outcomes of TAWDs. To achieve this, a thorough review and meta-analysis of current literature on this topic was carried out, focusing on acute versus delayed repair and mesh versus no mesh use.

## Materials and methods

### Search strategy

A broad and systematic search for all articles about TAWHs in PubMed, Embase, and the Cochrane Library was conducted by two reviewers (SK, RB) independently. The search syntax consisted of synonyms and MeSH/Emtree terms for traumatic abdominal wall hernias, as shown in Appendix 1 and Appendix 2. The literature search was not restricted to a certain period of time.

After removal of duplicates, all articles were screened on titles and abstracts, and irrelevant articles were excluded. After that, a full-text screening of the remaining articles was performed with predefined exclusion criteria. Studies that included outcome parameters were included; thus, studies that only reported on radiological findings were excluded, as well as studies without hernia recurrence rate as primary outcome. Moreover, articles were excluded when the full text was not available and language was limited to English, Dutch, or German. In case of disagreement of both reviewers, consensus was reached by discussion. All case series describing five patients or less were excluded as well.

### Methodological quality assessment

The selected articles were assessed on methodological quality by two reviewers independently (SK, RB), using a modified version of the CONSORT 2010 Checklist developed by the Consolidated Standards of Reporting Trials (CONSORT) Group [22]. Although this checklist was originally designed for reporting randomized controlled trials, it was adapted to make it appropriate for quality assessment of the selected articles. We also completed the Newcastle-Ottawa scale, which provides some more information on specific cohort studies [23]. In case of any doubt or disagreement, consensus was reached during an expert meeting.

### Data extraction

Data extraction was performed by two reviewers independently. The following data were extracted: first author, year and journal of publication, study design, country of study, number of TAWDs reported, size of surgical treatment groups, recurrence rates, and data about mortality and loss to follow-up. In particular, all articles were scrutinized for information on the timing of repair—whether this was performed during initial hospitalization or not—and the use of a mesh. Data on the location of the hernia was too scarce to encounter for further analysis.

### Definition of timing of hernia repair

The definitions of timing of repair differed between the included studies. Only Coleman et al. described well-defined periods of time. Hernia repair within 2 weeks post-trauma was classified as acute. The other four studies did not use these clear-cut definitions. Netto et al. and Honaker et al. used “acute” for repair during initial hospitalization, and “delayed” for all elective procedures any time afterwards. We used the definition of 2 weeks following the trauma as acute, and any time afterwards as delayed.

The corresponding authors of the included articles were contacted in case of insufficient or contradictory information.

### Hernia classifications

Throughout the past years, several TAWD classification systems have been developed, based on the mechanisms of injury [24, 25], or considering hernia characteristics such as size or location [26, 27]. More recently, Dennis et al. [2] proposed a comprehensive grading system based on the anatomical layers of the abdominal wall. This classification is used in this article, since it allows a description of the severity of injury to the abdominal wall musculature and potential herniation of abdominal contents (Appendix 3).

### Outcome measure

The main point of interest of this literature review was hernia recurrence, diagnosed either on physical examination or on computed tomography (CT) scans.

### Statistical analysis

All statistical analyses were performed using Review Manager 5 (RevMan 5, Cochrane Collaboration, Copenhagen, Denmark; 2014). Meta-analyses on hernia recurrence rates after mesh versus no mesh repair, and acute versus delayed repair were conducted separately. Heterogeneity amongst the included studies was assessed using the overall effect *Z*-test and additionally tau-squared (*T*^2^), chi-squared (*χ*^2^), degrees of freedom (df), and *I*^2^ statistical measures; all were calculated using the Review Manager program. Fixed effects were used since the *I*^2^ was equal to or less than 25% in all cases.

Because of computational difficulties in sub-analysis groups without hernia recurrences (“zero-cell counts”), the Mantel-Haenszel method was used to calculate odds ratios (OR) with 95% confidence intervals (95% CI) [28]. *P* values below 0.05 were considered statistically significant. 

## Results

### Search

**Fig. 1:**
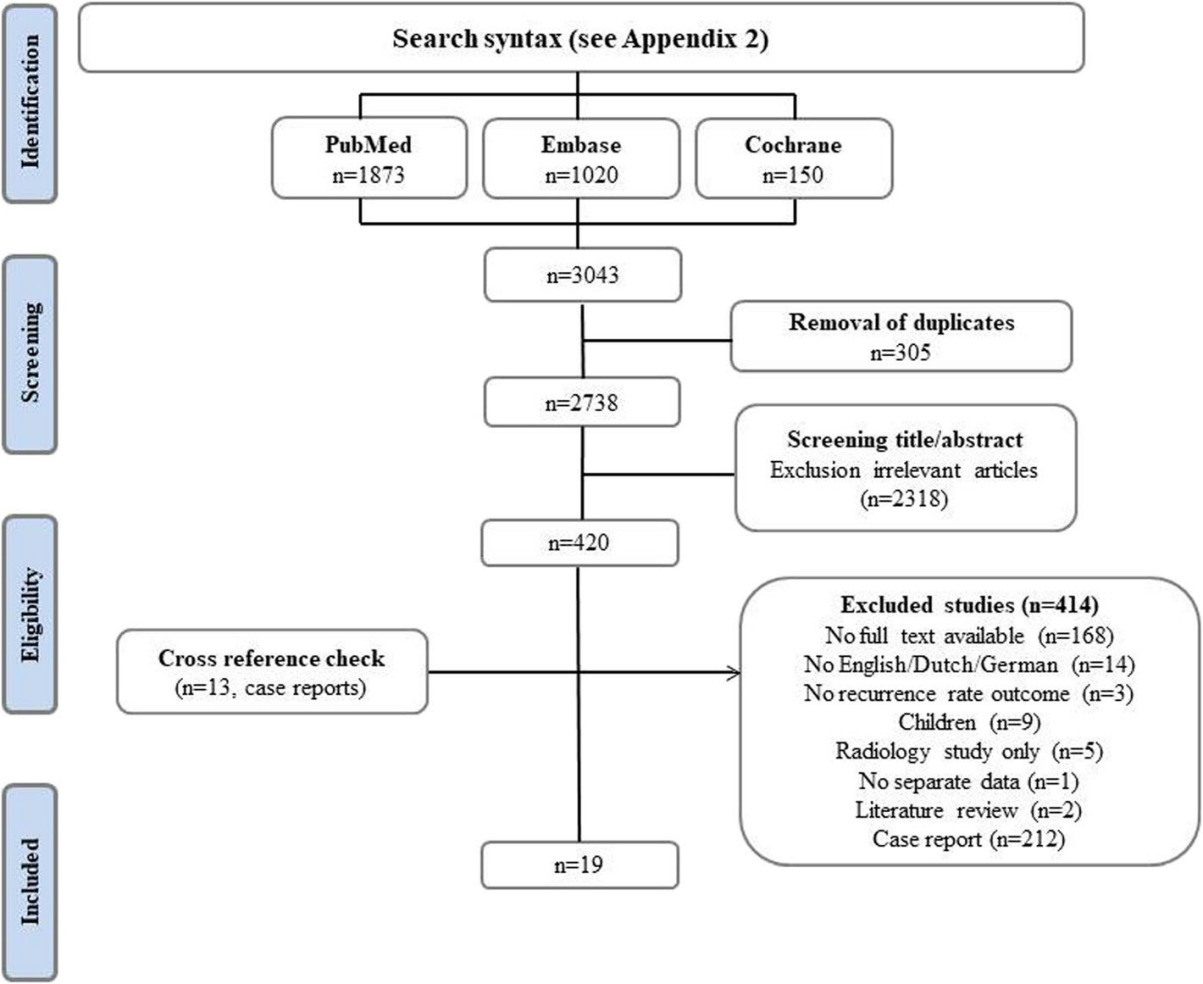
Screening and selection of the included studies. There were 3043 articles after the first search. After removal of 305 duplicates, 2738 articles remained. A total of 420 studies remained following title and abstract screening. Afterwards, 414 articles were excluded for several reasons, cross-reference checking only revealed case reports. In the end, only 19 studies remained

A search was performed at the end of December 2018 (Appendix 2). In total, 3043 articles were retrieved (Fig. 1). After removal of duplicates (*n* = 305) and exclusion on titles and abstracts (*n* = 2318), 420 remaining articles were full text assessed. Almost half of them were case reports and were excluded for this reason. Moreover, no full texts could be obtained from 168 articles. Three articles (Danto; Fullerton; Gupta et al. [11]) were not included because they did not account for recurrence rates. Finally, a total of 19 articles were selected. Cross-reference checking of this selection did not yield any other reviews on the management of TAWDs. As described previously in the methods section, all case series were excluded for final analysis to reduce heterogeneity. Hereafter, 6 articles remained for meta-analysis.

### Baseline characteristics

The baseline characteristics of the included studies are shown in Table 1. All selected articles had a retrospective study design and studied current practices in TAWD management and associated injuries requiring surgery. Moreover, all studies excluded patients with an abdominal wall defect after penetrating trauma, except for Park et al. [31] who included 9 patients with abdominal wall defects with 8 of them after blunt and 1 following penetrating trauma. Altogether they included a total of 273 patients with TAWDs.

**Table 1:** Baseline characteristics included studies

Author	Year	Journal	Country	Study period	Study population	TAWDs meeting inclusion criteria	TAWD type	Surgery (%)
Park	2018	Ann Surg Treat Res	Korea	2006–2015	9	8	All lumbar	8 (89)
Pardhan	2016	World J Surg	Australia	2003–2013	44	44	nm	41 (93)
Coleman	2015	J Trauma Acute Care Surg	USA	2002–2014	80	80	All types	23 (29)
Honaker	2014	J Trauma Acute Care Surg	USA	2007–2012	38	38	All types	30 (79)
Bender	2008	Am J Surg	USA	2001–2007	25	25	All types	22 (88)
Netto	2006	J Trauma	Canada	2000–2004	34	34	Mainly posterior	10 (29)
Vijayalakshmi [29]	2018	J Clin Diagn Res	India	nm	4	4	All types	4 (100)
Akbaba	2015	Indian J Surg	nm	nm	3	3	nm	2 (33)
Guttenridge	2014	ANZ J Surg	Australia	2007–2010	5	5	All types	4 (80)
Singal	2011	J Emerg Trauma Shock	India	nm	3	3	All types	3 (100)
Agarwal	2009	J Med Case Rep	India	nm	2	2	All types	2 (100)
Kumar	2004	Hernia	India	nm	2	2	All types	2 (100)
Burt	2004	J Trauma	USA	nm	3	3	Posterior	3 (100)
Brenneman	1995	J Trauma	Canada	1992–1993	9	9	All types	7 (78)
Damschen	1994	J Trauma	USA	nm	5	4	All types	2 (50)
Fullerton	1984	J Emerg Med	USA	nm	2	2	All types	2 (100)
Guly	1983	J Trauma	UK	nm	2	2	All types	2 (100)
Danto	1976	J Trauma	USA	nm	3	3	All types	3 (100)
Payne [30]	1973	J Trauma	USA	nm	2	2	All types	2 (100)

This table shows all the characteristics of the included studies. In most studies, the majority of patients are treated surgically for their TAWD except for Coleman, Netto, and Akbaba et al. The six studies on top of the table are included in the meta-analysis

*nm* not mentioned

### Methodological quality assessment

The included studies were critically appraised with a low inter-observer variation on their methodological quality using the predefined criteria, as summarized in Additional file 1 for the adjusted CONSORT checklist and Additional file 2 for the Newcastle-Ottawa scale. Overall, only Honaker et al. [4] study was graded with a high-moderate quality of evidence; the five other articles scored between moderate-low and very low in the CONSORT checklist, and with the Newcastle-Ottawa scale, all studies scored poor quality.

### Treatment of TAWD

In four of the studies that were included for analyses, most (> 75%) of the TAWDs were surgically repaired [3, 4, 18, 31]. Only the studies of Coleman et al. [17] and Netto et al. [1] reported low surgical repair rates of 29% both. All of the included studies reported on a diversity of reinforcement materials, including synthetic and biologic meshes. Besides these materials, Bender et al. [18] used acellular dermis of cadavers, and Brenneman et al. [19] reported on the use of autogenic tissue. Since Brenneman et al. [19] used various different treatment strategies with different muscle flaps to cover the defect, we did not include this study for further analysis on recurrence. 

### Type of repair

Only Honaker et al .[4]. accounted for the decision between primary and mesh repair. In this study, defects were repaired primarily when there was sufficient tissue to achieve a tension-free reconstruction. A mesh was used when a tension-free closure could not be accomplished, or when this was preferred by the surgeon over primary repair. The use of a mesh was only contraindicated in patients with abdominal contamination who had their defect repaired at initial surgery.

### Recurrence rates

**Fig. 2:**
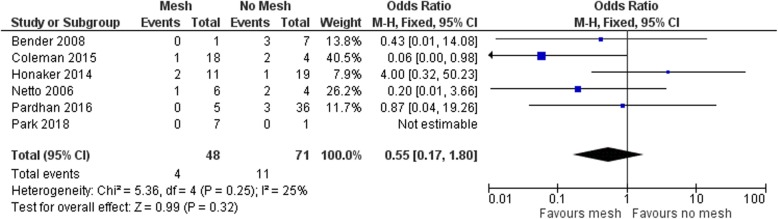
Hernia recurrence (mesh vs. no mesh). The odds ratio for hernia recurrence in the six studies included in the meta-analysis, revealing no significant difference for mesh or no mesh, with a total odds ratio of 0.55 and a 95% CI of 0.17–1.80

**Fig. 3:**
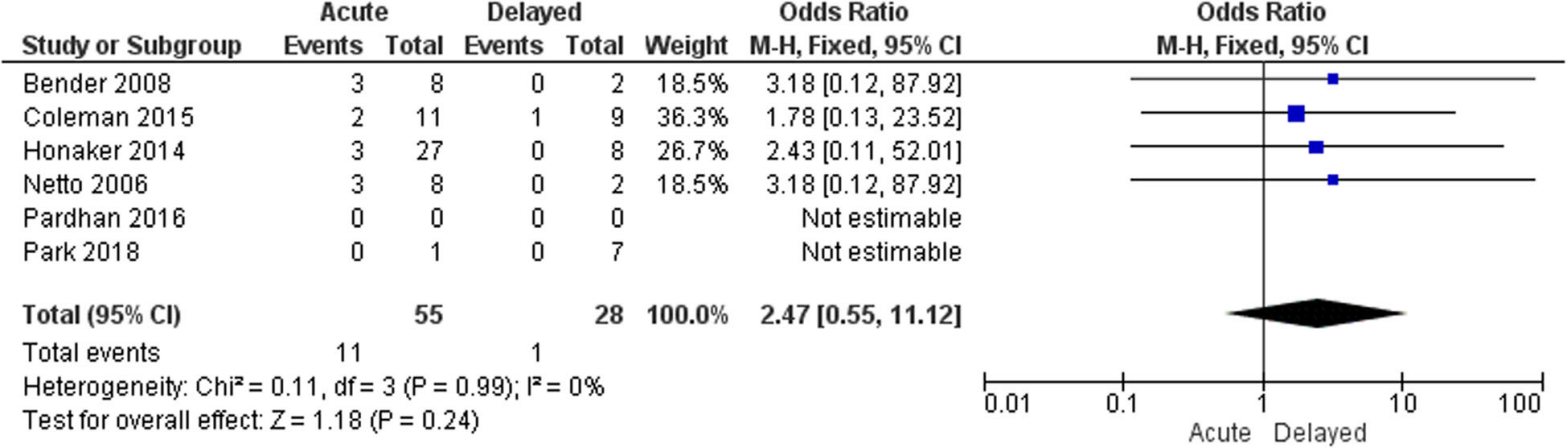
Hernia recurrence (acute vs. delayed repair). The odds ratios for hernia recurrence in patients following acute or delayed repair. No significant differences were found with a total odds ratio of 2.47 and a 95% CI of 0.55–11.12

Overall, no significant difference between the use of mesh and no mesh was seen in all included studies as shown in Fig. 2 (pooled OR 0.55 [95% CI 0.17–1.80]; *p* = 0.32). Similarly, none of the studies reported significant differences with regard to the timing of repair. No favor of acute or delayed repair was demonstrated, with an overall OR of 2.47 (95% CI 0.55–11.12; *p* = 0.24) (Fig. 3).

Twenty-three (13.5%) patients who underwent surgical repair showed recurrence (Table 2). The majority of them (*n* = 16.70%) had their TAWDs repaired without (mesh) reinforcement (Table 2). The other seven recurrences occurred in patients who had biologic meshes (*n* = 3), synthetic meshes (*n* = 2), or an absorbable mesh (*n* = 1) placed, with in one case no further description of the type of mesh.

**Table 2:** Outcome

Characteristics			Surgical repair				Outcome		
Author	Median ISS (range)	TAWDs	Total (%)	Acute	Delayed	Mesh	Recurrence rate (mesh used)	Mortality (%)	Loss to FU (%)
Park	nm	9	8 (89)	0	7	7	0	0	0
Pardhan	23 (nm)	44	41 (93)	8	33	5	3 (0)	4 (9)	1 (2)
Coleman	22 (nm)	80	23 (29)	18	5	7	6 (3)	0	nm
Honaker	17 (1–66)	38	30 (79)	27	3	11	3 (2)	2 (5)	0
Bender	35 (nm)	25	22 (88)	11	11	18*	3 (1)	1 (4)	3 (12)
Netto	31 (18–44)	34	10 (29)	8	2**	1	3 (0)	1 (3)	11 (32)
Vijayalakshmi	nm	4	4 (100)	4	0	0	0	0	0
Akbaba	nm	3	2 (33)	0	2	2	0	0	0
Guttenridge	14 (9–29)	5	4 (80)	3	1	2	0	0	1 (20)
Singal	nm	3	3 (100)	3	0	1	0	0	0
Agarwal	nm	2	2 (100)	2	0	1	0	0	0
Kumar	nm	2	2 (100)	2	0	0	0	0	0
Burt	nm	3	3 (100)	0	3	3	1 (1)	0	nm
Brenneman	nm (mean 25)	9	7 (78)	2	5	5	2 (0)	nm	nm
Damschen	nm	4	2 (50)	2	0	0	1 (0)	nm	2 (50)
Fullerton	nm	2	2 (100)	2	0	nm	0	1 (50)	nm
Guly	nm	2	2 (100)	1	1	0	0	0	0
Danto	nm	3	3 (100)	3	0	nm	nm	1 (33)	nm
Payne	nm	2	2 (100)	1	1	0	1 (0)	0	nm
Total	NA	274	172 (63)	98	74	63	23 (7)	10 (NA)	18 (NA)

This table shows surgical repair and outcome for all 19 studies. From a total of 274 patients with TAWDs, 172 underwent surgical repair, both acute (*n* = 98) and delayed (*n* = 74), with a minority of mesh repair (*n* = 63). A total of 23 recurrences occurred in this patient group

*nm* not mentioned, *NA* not applicable

*Reinforcement consists of absorbable mesh, permanent mesh, and acellular cadaver dermis

**Two of the patients who were initially treated conservatively developed symptoms requiring surgery after 8 months

Location of the hernia has not been mentioned in case of recurrence rates; therefore, there can be no conclusion on whether location of the hernia is relevant for recurrence.

## Discussion

This review of the literature shows that in most of the included patients who got a recurrence after surgical repair, the defect was repaired without mesh augmentation (70%) and during the acute posttraumatic period, defined as within 2 weeks after trauma. Despite this finding, based on our pooled analysis, neither a significant favor of mesh versus no mesh use, nor a significant difference between acute and delayed repair was demonstrated.

The current available literature on this topic is scarce. It is mainly limited to case reports and a few case series and literature reviews. We only found 6 retrospective studies which investigated current practices in TAWD management, including a total of 230 patients.

Most patients had their TAWDs repaired surgically, except for the studies conducted by Netto et al. [1] and Coleman et al. [17]. In these studies, only a small part of the study population (29%) underwent surgical repair. Based on their results, the authors advocated that operative exploration is not obligatory. They proposed conservative management in selected patients with asymptomatic defects who do not have associated injuries requiring urgent surgery [1, 17]. This recommendation, however, may be based on a low frequency of intra-abdominal injuries reported by Coleman et al. [17]. Moreover, 2 of the 26 patients who were initially managed non-operatively by Netto et al. [1] developed symptomatic defects and underwent secondary surgical repair. Ultimately, both studies did not classify the TAWDs based on the classification system provided by Dennis et al. [2]. Therefore, the severity of the injury to the abdominal wall musculature and—most important—potential herniation of abdominal contents could not be assessed.

When evaluating the results in the patient population that underwent surgery, two measures were studied: mesh use and timing of repair. Honaker et al. [4] noticed no recurrences at all in the patient group which underwent mesh repair. Nevertheless, four of the studies in our literature review did not report a significant favor for mesh or no mesh use, mainly due to a lack of power owing to small study sample sizes [1, 3, 4, 17]. Only Bender et al. [18] mentioned a significant difference; however, they used different reinforcements including absorbable meshes and the acellular dermis of cadavers, resulting in a heterogeneous comparison.

After all, repairing techniques according to current general principles (tension-free repair) are recommended to minimize recurrence rates [3, 18]. If this cannot be achieved primarily—this may be especially difficult in the acute posttraumatic period due to swelling and hematoma—a mesh should be used.

In addition to this, both Liasis et al. [16] and Bender et al. recommended mesh use in all patients with delayed TAWD repair, although they questioned mesh repair in emergency settings due to potential contamination. Contamination is considered to be a relative contra-indication for non-absorbable mesh use, due to an increased risk of infectious complications, eventually potentially mesh removal [3, 32–34].

Coleman et al. mentioned the use of open abdomen treatment for contamination in six patients following their primary surgery. Honaker et al. also mentioned an average number of 2.1 operations before TAWD repair in patients requiring damage control surgery at first.

In addition to contamination as a possible cause for delayed surgery, the physiological condition of the patient could also play a role in the decision to possibly postpone repair of the abdominal wall defect. Unfortunately, the information in most of the studies is limited, so these variables could not be included in our analysis.

Concerning the timing of repair, there was no significant difference between repair at initial trauma laparotomy or in an elective setting during hospitalization. By contrast, Honaker et al. [4] reported that all recurrences occurred in the group that underwent immediate repair. Moreover, Brenneman et al. [19], who repaired seven TAWDs at initial trauma laparotomy, observed that acute repair without mesh failed in a majority of the patients (5/7). Based on these results, it is not possible to make a strong recommendation for the optimal timing of repair though, since these studies were very heterogeneous in their description of timing of repair. Besides that, none of them demonstrated a significant difference between the acute and delayed repair groups. Ultimately, the choice for the timing of repair is mainly dependent on (the extent of) concomitant findings [16]. Early repair may be dictated by the extent of the injury and concomitant injuries, and therefore, the choice for management strategy must be based on individual circumstances [3, 17]. Moreover, as one may suggest, an early TAWD repair decreases the risk of bowel obstruction, strangulation, and/or incarceration [1, 5, 7, 9, 35].

To our knowledge, this is the second largest review on the treatment strategies of TAWDs. Since TAWDs are rare, there are only limited amounts of case reports, series, and reviews evaluating different treatment strategies. The first literature review was published in 2003 by Liasis et al. and included 145 articles with a total of 248 TAWD cases. However, this review included mainly case reports and case reviews, leading to a more heterogeneous study population.

Liasis et al. do propose a very helpful treatment algorithm in which all TAWDs are surgically repaired. Furthermore, they noted that timing and mesh repair is situation-dependent. They recommended mesh use in all patients with delayed TAWD repair, but they questioned mesh repair in emergency settings due to potential contamination. Concerning the timing of repair, in cases where neither an emergency laparotomy is required nor a risk of incarceration is present, Liasis et al. state that delayed repair is justified.

In comparison with Liasis et al., our literature review provides insight into a thorough search in current literature, excluding case reports and case series. This resulted in a more homogenous treated patient population. In our comprehensive review, it is noted that most of the recurrences occur in the patients following primary repair instead of with a mesh, even though this does not show in the pooled analysis.

Timing does not seem to be an important predictor for recurrence, making a patient’s physiology (condition and concomitant injuries) the most important factor in determining the timing of repair.

There are several limitations to this study. First of all, these results are still based on relatively small numbers of patients. Therefore, the recommendations regarding optimal timing of repair and mesh use should be interpreted carefully. The variation in follow-up duration (1–60 months) across the selected studies also affected the outcome of the pooled results. Secondly, regarding the methodological quality of the included studies, only Honaker et al. [4] was graded with a high-moderate quality of evidence; the five other articles scored between moderate-low and very low. Besides that, the selected articles did neither account extensively for concomitant injuries nor the size of the defect and presence of herniation. Moreover, differences in definitions of timing of repair and a variety of mesh types led to a heterogeneous study population, possibly leading to selection bias. In addition to this, the risk of bias has been increased by Netto et al. who reported a striking loss to follow-up (32%). Lastly, a lack of information on the choice for the treatment strategy (primary versus mesh repair; acute versus delayed repair) made it impossible to propose a treatment algorithm.

## Conclusion

Although not statistically significant in a pooled analysis, it is noted that 70% of the recurrences were found in patients without the use of mesh augmentation during repair. Furthermore, it is important to note that timing is not an important risk factor for recurrence, which makes the choice for the timing of repair highly dependent on the individual circumstances of each patient. Most patients have multiple concomitant injuries, but no increased risk was found for early repair after trauma. A larger, multicenter prospective cohort study is required to evaluate recurrence rates after TAWD repair.

### Supplementary information

**Additional file 1.** Methodological quality assessment—CONSORT checklist.

**Additional file 2.** Predefined criteria on methodological quality assessment.

## Data Availability

Data sharing not applicable to this article as no datasets were generated or analyzed during the current study.

## Search terms

TAWH

TAWHs

Handlebar hernia

Handlebar hernias

Handlebar herniation

Handlebar herniations

Handlebar injury

Handlebar injuries

Handlebar trauma

Trauma

Wounds and injuries [MESH terms]

Traumatic

Posttraumatic

Seat belt

Seat belts [MESH terms]

Seatbelt

Seatbelts

Seat-belt

Seat-belts

Abdominal wall

Abdominal wall [MESH terms]

Abdominal-wall

Abdominal fascia

Abdominal muscle

Abdominal muscles

Abdominal muscles [MESH terms]

Abdominal musculature

Abdominal wall musculature

Hernia

Hernias

Hernia, abdominal [MESH terms]

Hernia, ventral [MESH terms]

Hernia, inguinal [MESH terms]

Herniation

Herniations

Avulsion

Avulsions

Defect

Defects

Disruption

Disruptions

Injury

Injuries

Rupture

Ruptures

Hernia [MESH terms]

## Appendix 2

**Table 3:** Search syntax

Database	Syntax	Hits
Pubmed (31-12-2018)	**#1 OR (#2 AND #3 AND #4)** **#1:** ((((((((TAWH[Title/Abstract]) OR TAWHs[Title/Abstract]) OR “Handlebar hernia”[Title/Abstract]) OR “Handlebar hernias”[Title/Abstract]) OR “Handlebar herniation”[Title/Abstract]) OR “Handlebar herniations”[Title/Abstract]) OR “Handlebar injury”[Title/Abstract]) OR “Handlebar injuries”[Title/Abstract]) OR “Handlebar trauma”[Title/Abstract] **138 hits** **#2:** (((((((((Trauma[Title/Abstract]) OR (wounds and injuries[MeSH Terms])) OR Traumatic[Title/Abstract]) OR Posttraumatic[Title/Abstract]) OR “Seat belt”[Title/Abstract]) OR seat belts[MeSH Terms]) OR Seatbelt[Title/Abstract]) OR Seatbelts[Title/Abstract]) OR “Seat-belt”[Title/Abstract]) OR “Seat-belts”[Title/Abstract] **955.720 hits** **#3:** ((((((((“Abdominal wall”[Title/Abstract]) OR abdominal wall[MeSH Terms]) OR “Abdominal-wall”[Title/Abstract]) OR “Abdominal fascia”[Title/Abstract]) OR “Abdominal muscle”[Title/Abstract]) OR “Abdominal muscles”[Title/Abstract]) OR abdominal muscles[MeSH Terms]) OR “Abdominal musculature”[Title/Abstract]) OR “Abdominal wall musculature”[Title/Abstract] **33.657 hits** **#4:** (((((((((((((((((Hernia[Title/Abstract]) OR Hernias[Title/Abstract]) OR hernia, abdominal[MeSH Terms]) OR hernia, ventral[MeSH Terms]) OR hernia, inguinal[MeSH Terms]) OR Herniation[Title/Abstract]) OR Herniations[Title/Abstract]) OR Avulsion[Title/Abstract]) OR Avulsions[Title/Abstract]) OR Defect[Title/Abstract]) OR Defects[Title/Abstract]) OR Disruption[Title/Abstract]) OR Disruptions[Title/Abstract]) OR Injury[Title/Abstract]) OR Injuries[Title/Abstract]) OR Rupture[Title/Abstract]) OR Ruptures[Title/Abstract]) OR Hernia[MeSH Terms] **1.238.152 hits**	1873
Embase (31-12-2018)	**(#1 OR (#2 AND #3 AND #4)) AND [embase]/lim** **#1:** ‘traumatic abdominal wall hernia’:ab,ti OR ‘traumatic abdominal wall hernias’:ab,ti OR tawh:ab,ti OR tawhs:ab,ti OR ‘handlebar hernia’:ab,ti OR ‘handlebar hernias’:ab,ti OR ‘handlebar herniation’:ab,ti OR ‘handlebar herniations’:ab,ti **165 hits** **#2:** trauma:ab,ti OR traumatic:ab,ti OR posttraumatic:ab,ti OR handlebar:ab,ti OR ‘seat belt’:ab,ti OR ‘seat belts’:ab,ti OR seatbelt:ab,ti OR seatbelts:ab,ti OR ‘seat-belt’:ab,ti OR ‘seat-belts’:ab,ti **435.730 hits** **#3:** ‘abdominal wall’:ab,ti OR ‘abdominal wall’/exp OR ‘abdominal-wall’:ab,ti OR ‘abdominal fascia’:ab,ti OR ‘abdominal muscle’:ab,ti OR ‘abdominal muscles’:ab,ti OR ‘abdominal musculature’:ab,ti OR ‘abdominal wall musculature’/exp **47.282 hits** **#4:** hernia:ab,ti OR hernias:ab,ti OR ‘abdominal wall hernia’/exp OR herniation:ab,ti OR herniations:ab,ti OR defect:ab,ti OR defects:ab,ti OR disruption:ab,ti OR disruptions:ab,ti OR injury:ab,ti OR injuries:ab,ti OR rupture:ab,ti OR ruptures:ab,ti **1.666.090 hits**	1020
Cochrane (31-12-2018	**#1 OR (#2 AND #3 AND #4)** **#1:** tawh:ti,ab OR tawhs:ti,ab OR “handlebar hernia”:ti,ab OR “handlebar hernias”:ti,ab OR “handlebar herniation”:ti,ab OR “handlebar herniations”:ti,ab OR “handlebar injury”:ti,ab OR “handlebar injuries”:ti,ab OR “handlebar trauma”:ti,ab **0 hits** **#2:** trauma:ti,ab or traumatic:ti,ab or posttraumatic:ti,ab or handlebar:ti,ab or “seat belt”:ti,ab or “seat belts”:ti,ab or seatbelt:ti,ab or seatbelts:ti,ab or seat-belt:ti,ab or seat-belts:ti,ab **17.029 hits** **#3:** “abdominal wall”:ti,ab or abdominal-wall:ti,ab or “abdominal fascia”:ti,ab or “abdominal muscle”:ti,ab or “abdominal muscles”:ti,ab or “abdominal musculature”:ti,ab or “abdominal wall musculature”:ti,ab or “anterior abdominal”:ti,ab or flank:ti,ab or lumbar:ti,ab or inguinal:ti,ab or groin:ti,ab or spigeli:ti,ab or spigelian:ti,ab **15.442 hits** **#4:** hernia:ti,ab or hernias:ti,ab or herniation:ti,ab or herniations:ti,ab or defect:ti,ab or defects:ti,ab or disruption:ti,ab or disruptions:ti,ab or injury:ti,ab or injuries:ti,ab or rupture:ti,ab or ruptures:ti,ab **45.835 hits**	150

## Appendix 3

**Table 4:** TAWD classification system (Dennis et al. [2])

TAWH classification system	
Grade I	Subcutaneous tissue contusion
Grade II	Abdominal wall muscle hematoma
Grade III	Single abdominal muscle disruption
Grade IV	Complete abdominal wall muscle disruption
Grade V	Complete abdominal wall muscle disruption with herniation of abdominal contents
Grade VI	Open herniation (evisceration)

## Abbreviations

CI: Confidence interval
CT: Computed tomography
OR: Odds ratio
TAWD: Traumatic abdominal wall defect
