# Supplementary material for: Timing of repair and mesh use in traumatic abdominal wall defects: a systematic review and meta-analysis of current literature
Source: World J Emerg Surg. 2019 Dec 17;14:59. doi: 10.1186/s13017-019-0271-0 (PMC6918711; doi:10.1186/s13017-019-0271-0)
Supplement: Supplementary file 1 — Additional file 1. Methodological quality assessment—CONSORT checklist. [file 13017_2019_271_MOESM1_ESM.docx]

**Additional file 1a. Methodological Quality Assessment – CONSORT checklist**

|  | **Study** | **Pardhan (2016)** | **Coleman (2015)** | **Honaker (2014)** | **Bender**  **(2008)** | **Netto**  **(2006)** | **Park**  **(2018)** |
| --- | --- | --- | --- | --- | --- | --- | --- |
| **Methods** | **Study design** |  |  |  |  |  |  |
|  | **Participants** |  |  |  |  |  | Not applicable |
|  | **Intervention strategy** |  |  |  |  |  |  |
|  | **Outcome** |  |  |  |  |  |  |
|  | **Statistical methods** |  |  |  |  |  |  |
| **Results** | **Participant flow** |  |  |  |  |  |  |
|  | **Recruitment** |  |  |  |  |  |  |
|  | **Baseline data** |  |  |  |  |  |  |
|  | **Intervention** |  |  |  |  |  |  |
|  | **Outcome** |  |  |  |  |  |  |
|  | **Additional analyses** |  |  |  | Not applicable |  |  |
|  | **Complications** |  |  |  |  |  |  |
| **Discussion** | **Limitations** |  |  |  |  |  |  |
|  | **Generalizability** |  |  |  |  |  |  |
|  | **Interpretation** |  |  |  |  |  |  |
|  | **Overall quality score** | Moderate-low | Low | High-moderate | Moderate-low | Very low | Low |

**Additional file 1b. Predefined Criteria Methodological Quality Assessment - Methods**

| METHODS | |
| --- | --- |
| Study design | |
|  | Prospective cohort study |
|  | Retrospective cohort study; participants included after screening all admitted patients who sustained blunt trauma |
|  | Retrospective cohort study; not screening all hospital admissions after blunt trauma |
| Participants | |
|  | Setting(s) and location(s) of data collection are well described *and* eligibility criteria of participants are mentioned |
|  | Setting(s) and location(s) of data collection are well described *or* eligibility criteria of participants are mentioned |
|  | None of the above |
| Intervention strategy | |
|  | Described with sufficient details allowing replication, including timing *and* method of repair |
|  | Described with sufficient details allowing replication, including method of repair |
|  | Not or inadequately described to allow replication |
| Outcome | |
|  | Well defined pre-specified outcome measure(s) |
|  | Moderately defined pre-specified outcome measure(s) |
|  | No pre-specified outcome measure(s) mentioned |
| Statistical methods | |
|  | Adequate description of statistical methods used to compare groups for outcome measures, including methods for additional analyses |
|  | Moderate description of statistical methods used to compare groups for outcome measures, without methods for additional analyses |
|  | Inadequate description of statistical methods used |

**Additional file 1c. Predefined Criteria Methodological Quality Assessment - Results**

| RESULTS | |
| --- | --- |
| Participant flow | |
|  | Numbers of patients who were surgically treated and were analyzed for the primary outcome, including deaths and losses with reasons (when applicable) |
|  | Numbers of patients who were surgically treated and were analyzed for the primary outcome, without reasons for deaths or losses |
|  | None of the above |
| Recruitment | |
|  | Periods of recruitment *and* follow-up defined |
|  | Follow-up time defined, but inadequately/not accounting for the recruitment period |
|  | None of the above |
| Baseline data | |
|  | Adequate description of baseline patient characteristics, including ISS *and* concomitant intra-abdominal injuries |
|  | Adequate description of baseline patient characteristics, but without data on ISS *or* concomitant intra-abdominal injuries |
|  | None of the above |
| Intervention | |
|  | Described with sufficient details, including numbers, timing *and* method of repair |
|  | Described with sufficient details, including numbers *and* method of repair |
|  | Inadequately described to allow replication or inconsistency |
| Outcome | |
|  | Adequate description of recurrence rates, including information on timing (acute versus delayed) *and* method of repair (mesh versus no mesh) |
|  | Adequate description of recurrence rates, including information on timing (acute versus delayed) *or* method of repair (mesh versus no mesh) |
|  | None of the above |
| Additional analyses | |
|  | Adequate description of results of additional analyses performed, including subgroup analyses, distinguishing pre-specified from exploratory |
|  | Adequate description of results of additional analyses performed, including subgroup analyses, not distinguishing pre-specified from exploratory |
|  | None of the above |
| Complications | |
|  | Adequate description of important complications, including patient characteristics *and* necessary re-admissions/re-operations |
|  | Moderate description of important complications, without information about necessary re-admissions |
|  | None of the above |

**Additional file 1d. Predefined Criteria Methodological Quality Assessment – Discussion**

| DISCUSSION | |
| --- | --- |
| Limitations | |
|  | Mentioning study limitations *and* sources of potential bias |
|  | Mentioning study limitations *or* sources of potential bias |
|  | None of the above |
| Generalizability | |
|  | Adequately accounting for external validity of the findings |
|  | Moderately accounting for external validity of the findings |
|  | Not or insufficient accounting for external validity of the findings |
| Interpretation | |
|  | Adequate interpretation and conclusion, consistent with results, *and* considering other relevant evidence |
|  | Moderate interpretation and conclusion, consistent with results |
|  | None of the above |
