# Supplementary material for: Timing of repair and mesh use in traumatic abdominal wall defects: a systematic review and meta-analysis of current literature
Source: World J Emerg Surg. 2019 Dec 17;14:59. doi: 10.1186/s13017-019-0271-0 (PMC6918711; doi:10.1186/s13017-019-0271-0)
Supplement: Supplementary file 2 — Additional file 2. Predefined criteria on methodological quality assessment. [file 13017_2019_271_MOESM2_ESM.docx]

**Additional file 2 – Methodological quality assessment**

NEWCASTLE - OTTAWA QUALITY ASSESSMENT SCALE COHORT STUDIES

| **Study** | **Park** | **Pardhan** | **Coleman** | **Honaker** | **Bender** | **Netto** |
| --- | --- | --- | --- | --- | --- | --- |
| **Selection** | | | | | | |
| Representativeness exposed cohort | * | * | * | * | * | * |
| Selection non exposed | - | - | - | - | - | - |
| Ascertainment exposure | * | * | * | * | * | * |
| Outcome not present at start | * | * | * | * | * | * |
| **Comparability** | | | | | | |
| Comparability on basis of design or analysis | - | - | - | - | - | - |
| **Outcome** | | | | | | |
| Assessment of outcome | * | * | * | * | * | * |
| Follow-up long enough | - | * | * | * | * | - |
| Adequacy of follow-up | * | * | - | * | * | - |
| ***Overall quality score*** | Poor | Poor | Poor | Poor | Poor | Poor |

**Additional file 2b. Predefined criteria on methodological quality assessment**

**Selection**

1) Representativeness of the exposed cohort

a) Truly representative of the average _______________ (describe) in the community ****

b) Somewhat representative of the average ______________ in the community ****

c) Selected group of users eg nurses, volunteers

d) No description of the derivation of the cohort

2) Selection of the non exposed cohort

a) Drawn from the same community as the exposed cohort ****

b) Drawn from a different source

c) No description of the derivation of the non exposed cohort

3) Ascertainment of exposure

a) Secure record (eg surgical records) ****

b) Structured interview ****

c) Written self report

d) No description

4) Demonstration that outcome of interest was not present at start of study

a) Yes ****

b) No

**Comparability**

1) Comparability of cohorts on the basis of the design or analysis

a) Study controls for _____________ (select the most important factor) ****

b) Study controls for any additional factor **** (This criteria could be modified to indicate specific control for a second important factor.)

**Outcome**

1) Assessment of outcome

a) Independent blind assessment ****

b) Record linkage ****

c) Self report

d) No description

2) Was follow-up long enough for outcomes to occur

a) Yes (select an adequate follow up period for outcome of interest) ****

b) No

3) Adequacy of follow up of cohorts

a) Complete follow up - all subjects accounted for ****

b) Subjects lost to follow up unlikely to introduce bias - small number lost - > ____ % (select an adequate %) follow up, or description provided of those lost) ****

c) Follow up rate < ____% (select an adequate %) and no description of those lost

d) No statement

**Overall score**

Good quality: 3 or 4 stars in selection domain AND 1 or 2 stars in comparability domain AND 2 or 3 stars in outcome/exposure domain

Fair quality: 2 stars in selection domain AND 1 or 2 stars in comparability domain AND 2 or 3 stars in outcome/exposure domain

Poor quality: 0 or 1 star in selection domain OR 0 stars in comparability domain OR 0 or 1 stars in outcome/exposure domain
